# Challenges of Justice in the Context of Plant Genetic Resources

**DOI:** 10.3389/fpls.2019.01266

**Published:** 2019-10-25

**Authors:** Anna Deplazes-Zemp

**Affiliations:** Ethics Research Institute (ERI), Centre for Ethics of the University of Zurich, Zurich, Switzerland

**Keywords:** access and benefit-sharing, commutative justice, Convention on Biological Diversity, distributive justice, environmental justice, genetic resources

## Abstract

In this article, I discuss access and benefit-sharing (ABS) for plant genetic resources from an ethical perspective. This leads to the question of what types of justice actually play a role when more equity and fairness is demanded for plant genetic resources. Five dimensions of justice will be distinguished: classical distributive justice, which deals with a fair distribution of goods; commutative justice, which concerns a fair exchange of “give-and-receive”; justice as recognition, which relates to treating all involved parties with the same respect; reparative justice, which pertains to fair amendments for wrongful actions in the past; and procedural justice, which is concerned with just decision processes. Drawing on the discussion of ethical problems with biopiracy, the distribution of environmental burdens, and plant genetic resources in agriculture, I will illustrate that the use of genetic resources poses challenges across all five dimensions of justice. Because the combination of justice challenges is specific for each case of resource use, I will argue that it is important that users of genetic resources are aware of the complexity of justice problems to ensure fair and equitable ABS negotiations.

“Justice” is a paramount ideal underlying the debates on how to regulate and use plant genetic resources. We discuss questions such as *Who has rights to access and use these resources? How should benefits be shared? How should the use of genetic resources be regulated?* *Who should be involved in discussing these questions?* All of these are questions concerning justice. They are asked and discussed with the aim of finding answers that take into account what is due to all those who have a stake in genetic resources. I have written this overview article from an understanding of justice in analytic philosophy.^1^ Although there is wide agreement that justice is important, there may be different views on what it means to safeguard justice in the development of policy as well as in specific access and benefit sharing negotiations. One reason for such disagreement is that justice is a concept with different dimensions. I will distinguish between five such dimensions, which all play a role in dealing with genetic resources. The aim of this analysis is to contribute to the understanding of first, why the use of genetic resources generates so much attention and controversy, and second, what needs to be considered in regulating and handling them justly. By discussing these issues, I am addressing not only philosophers but also an interdisciplinary readership, hoping that the article provides an occasion for them to take a step back from the everyday occupation with genetic resources and to reflect on the ethical implications associated with their use. Ideally, this will contribute toward bringing more justice reflections into the drafting or implementing of regulatory schemes. Moreover, these reflections may facilitate specific access and benefit-sharing (ABS) negotiations, which may be complicated by the fact that the different parties are prioritizing different dimensions of justice possibly without awareness of this source of disagreement.

After a brief introduction of the conception of justice underlying this article, each of the five dimensions of justice will be presented separately. For this purpose, I will start with a general introduction of the particularities of the respective justice dimension followed by a discussion of the role that it plays in the context of genetic resources. In doing so, I will draw on my own previous research and connect it to the philosophical work of other authors, for instance, Bram de Jonge and Doris Schroeder. Moreover, I will connect the literature on justice for genetic resources with parallel discourses on environmental justice or restorative justice. The presentation of the five dimensions of justice will be followed by the discussion of three practical justice challenges to illustrate how the justice dimensions meet: first, biopiracy; second, the distribution of environmental burdens; and third, plant genetic resources in agriculture. The article will close with two practical conclusions for a fair and equitable use of plant genetic resources.

## Five Dimensions of Justice in Dealing With Genetic Resources

The three most influential international treaties that introduced ABS for genetic resources are the Convention on Biological Diversity (CBD),^2^ the Nagoya Protocol (NP),^3^ and the International Treaty on Plant Genetic Resources for Food and Agriculture (ITPGRFA).^4^ These treaties have the explicit objective to ensure “fair and equitable sharing of benefits” from genetic resources. In spite of the prominence of the clause “fair and equitable,” for instance, in the full title of the Nagoya Protocol, and notwithstanding the long tradition that this clause has in investment treaties (Dolzer, 2005), its meaning remains undefined and vague (De Jonge, 2011; Vermeylen and Walker, 2011; Morgera, 2015). It is evident, however, that in one way or another fair and equitable benefit-sharing aims at introducing justice into the regulation and use of genetic resources. In that sense, Bram de Jonge discusses the question, *What is fair and equitable benefit-sharing?* by analyzing different principles of justice (De Jonge, 2011), and Morton Walloe Tvedt and Tomme Young also raise mainly justice topics in their analysis of the meaning of “fair and equitable” in ABS (Tvedt and Young, 2007: pp. 83–91).

For the purpose of this article, I assume that justice is a morally weighty demand, and I start from a very basic and general meaning of “justice” going back to the Roman law, where it was defined in the Institutes of Justinian as “the set and constant purpose which gives to every man his due” (Justinian, 1913: Book I, Title I). In a modern view, influenced by Enlightenment philosophy, this means that justice is based on the acknowledgment that all human beings have equal moral status and basic moral rights. I will distinguish between five different dimensions of justice: distributive justice, which deals with a fair distribution; commutative justice, which concerns a fair exchange of give-and-receive; justice as recognition, which relates to treating all involved parties with the same respect; reparative justice, which pertains to fair amendments for wrongful actions in the past; and procedural justice, which is concerned with just decision processes (For an overview, see **Table 1**). Each of these justice dimensions takes up other aspects of what it means to give everybody their due. The aim of the following introduction of the five dimensions of justice is not to suggest what a just ABS system for genetic resources would look like. Instead, I attempt to contribute to a better understanding of the challenges at hand and the reasons why there is controversy surrounding how to solve them.^5^

**Table 1:** Comparing the different justice dimensions when justice in general means that everybody gets his or her due.

Justice dimension	
Distributive justice	To give everybody their due shares in benefits and costs.
Commutative justice	To give everybody the due compensation in exchange for a good or service that was provided.
Justice as recognition	To give everybody their due respect.
Reparative justice	To give due redress to those who suffered injustice and possibly due punishment to those who committed it.
Procedural justice*	To give everybody their due voice and participation in decision making processes.

*Procedural justice-le is defined in the narrow sense used in the environmental justice discourse.

Let me start now with the presentation of the five dimensions of justice, which refer to different aspects of what we owe to others.

## Distributive Justice

Distributive justice is concerned with fair distribution of, for instance, material goods, such as natural resources. A large body of literature is dedicated to the question of who has rights to own, control, or benefit from natural resources (e.g., Nine, 2012; Armstrong, 2017). Key questions in that discourse are to what extent nation states have exclusive rights to benefits from natural resources that are found on state territory, and whether or how all people around the globe should be able to benefit at least partially from those resources. Besides natural resources, the distribution of non-material goods, such as power or opportunities, has also received wide attention in political philosophy (Rawls, 1971; Dworkin, 2000). Although distributive justice can be based on the idea that a just distribution provides an equal share of the distributed good to each party, there are also alternative views. John Rawls, for instance, famously suggested a “difference principle” which is based on a maximin criterion, meaning that unequal distribution of income or wealth can be just if this distribution still leads to advantages for the worst-off party compared with the available alternatives (Rawls, 1971).

In addition to the importance of a fair distribution of *goods*, more recently the demand for distributive justice has also been expressed regarding global and local *bads*. Distributive justice in this sense is one central pillar of the concept of “environmental justice” that goes back to a grassroots movement in the 1990s in the United States. The movement pointed to the existing environmental injustice when environmental risks and burdens, such as waste dumpsites, toxic emissions or other contaminations, are unequally distributed. Some communities endure more environmental burdens than others, and often, it is previously disadvantaged, marginalized, or impoverished groups that, in addition to these social injustices, also suffer environmental injustice (Bullard, 1993; Schlossberg, 2007; Walker, 2012).

### Distributive Justice for Genetic Resources

Distributive justice is one of the most widely discussed justice dimensions in the literature on genetic resources (e.g. De Jonge and Korthals, 2006; Korthals and De Jonge, 2009; Schroeder and Pogge, 2009; Schroeder and Pisupati, 2010; Vermeylen and Walker, 2011; Deplazes-Zemp, 2019). The term “genetic resources” suggests that they are a type of natural resource, a view also supported by the current regulatory framework in which genetic resources are placed under state sovereignty over natural resources. The understanding of genetic resources as a type of natural resource directly links this discussion to the literature on resource rights (Deplazes-Zemp, 2019). However, to treat genetic resources analogously to other natural resources may be problematic because genetic resources are a very particular type of natural resource. They can be described as atypical with respect to first, their non-tangible nature; second, the close connection between natural and cultural formation; as well as third, their connection to biodiversity and its vulnerability (Deplazes-Zemp, 2018b). These three features of genetic resources are relevant when it comes to distributive justice (Deplazes-Zemp, 2019), therefore, I will explain each of them in some more detail. It has been highlighted that genetic resources are non-tangible and that they carry information (Vogel, 1994; Millum, 2010; De Jonge, 2011; Tvedt and Schei, 2013; Ruiz Muller, 2015; Deplazes-Zemp, 2018b). I thus speak of the “informational” nature of these resources and suggest that this particular feature can best be illustrated by contrasting genetic resources with other biological resources such as timber or fish. While benefits from the latter are material and used as food or building material, it is the information in genetic resources that is of value among other things because it can lead to the generation of new material outside the provider country. According to such an interpretation of genetic resources it is thus the information that is exported from the country of origin and used and propagated, for instance in breeding processes or in biological or chemical procedures (Vogel, 1994; De Jonge, 2011; Ruiz Muller, 2015; Deplazes-Zemp, 2018b).^6^ The informational nature implies that territorial claims of countries of origin are more difficult to legitimize for genetic resources than for material natural resources. While in the case of material resources a constant supply from the country of origin is required for their use, in the case of genetic resources, only a one-time extraction of a small material sample is needed. Therefore, the territorial connection of the latter type of resource is weaker.

Although the question of how to share *material* or *financial* benefits from genetic resources is central to the debate, the Annex of the NP also lists a variety of potential *non-monetary* benefits from genetic resources that could be shared and that could be understood as a distribution of opportunities. The list includes benefits such as research collaboration, admittance to databases, access to scientific information, capacity-building and training.^7^ In addition, Bram de Jonge and Michiel Korthals suggest that “upstream benefit-sharing” should also be taken into account for distributive justice; by this, they mean opportunities and power to influence research and development agendas in the context of genetic resources (De Jonge and Korthals, 2006; Korthals and De Jonge, 2009). The authors suggest that this distributive aspect should be considered in decision making procedures for different uses of genetic resources, which directly relates to what I will discuss later in the sections on justice as recognition and procedural justice.

Finally, the third atypical feature of genetic resources is that they are related to biodiversity through the diversity of species that actually or potentially carry genetic resources. Biodiversity could also be the source of evolution of novel genetic resources in the future, and it is the condition for intact ecosystems in which current genetic resources prosper. This connection implies that genetic resources are vulnerable to decimation and extinction of biodiversity. In contrast to many other natural resources and because of their informational nature, genetic resources are not endangered by over-exploitation but by other environmentally destructive practices against which they must be actively protected. It can thus be argued that distributive justice for genetic resources concerns not only the distribution of benefits but also the costs for biodiversity protection. In other words, not only goods but also bads associated with genetic resources should be distributed fairly (Deplazes-Zemp, 2019).

## Commutative Justice

Commutative justice is also called “justice in exchange” because it concerns a fair exchange of items or services with the goal of achieving equivalence between giving and receiving (Schroeder and Pogge, 2009; Schroeder and Pisupati, 2010; De Jonge, 2011; Deplazes-Zemp, 2018a). In situations where goods are not exchanged for other goods but where products or services are exchanged for money, this leads to the discussion of just prices or just compensation, and is thus an important principle in the economic context (Koslowski, 2001). The “Fairtrade” label, for instance, symbolizes the aim of generating more commutative justice in economic exchange between farmers in low-income countries and companies in the industrialized world. One of the great challenges in this justice dimension is to determine under what conditions both sides contribute equivalents to the exchange if they are of a different nature. To deal with these substantial difficulties, commutative justice is sometimes also understood in a more procedural sense, according to which an exchange is considered just if both parties voluntarily consent to the transaction procedure employed (Schroeder and Pisupati, 2010). In this case, an overstated price could be considered to be just as long as the buying party pays it voluntarily. Such an interpretation of commutative justice overlaps with the dimension of procedural justice that this article addresses later.

### Commutative Justice for Genetic Resources

An ABS system for genetic resources can be understood as a mechanism to deal with the demands of commutative justice. ABS regulates how benefits should be shared *in exchange for* access to genetic resources (Schroeder and Pogge, 2009; Schroeder and Pisupati, 2010; De Jonge, 2011; Deplazes-Zemp, 2018a).^8^ The existing focus on the commutative aspect in ABS has been criticized not only because it cannot account for the complexity of justice issues at stake but also because it introduces a clear separation between providers and users of genetic resources. Critics highlight that this does not reflect the real world, where genetic resources are also being used in so-called provider countries (particularly threshold countries) and where it is thus not possible to draw a clear line between the two categories (Korthals and De Jonge, 2009; Nijar et al., 2016). Moreover, it again seems to be the informational nature of genetic resources that complicates the application of commutative justice schemes, which have been developed for material goods. Although it may be relatively straightforward to determine the provider of a material natural resource, such as a barrel of petrol, this is much more difficult in the case of genetic resources, which may have travelled a long distance in the form of a small material sample, an extract or even as a digital sequence before they are actually being used. This means that not only must resource access be controlled for actual users but any removal of minimal quantities of genetic resources, even for non-commercial purposes, must be monitored because they might be used as resources in the future (De Jonge, 2011). I suggested elsewhere that the informational nature of genetic resources is also responsible for another difficulty with ABS achieving commutative justice. We need to ask ourselves what it is that is actually being exchanged in ABS, who has claims on the exchanged goods, and how the claimant can be appropriately compensated (Deplazes-Zemp, 2018a). Does the provider state from the territory of which, for instance, a plant sample has been extracted, really have particular claims on this plant as a genetic resource? It is certainly true that the users of genetic resources gain from information in nature and it seems to be a legitimate request that they give something back in exchange for these free benefits. However, one may wonder whether provider states really are the appropriate recipients of such compensation or whether it should go, for example, directly to biodiversity protection projects, which preserved valuable genetic resources.^9^

Another interesting topic with regard to commutative justice is ABS for traditional knowledge associated with the use of genetic resources. If, for instance, a company uses the knowledge of an indigenous community about a particular health benefit of a plant, this knowledge is also subject to access and benefit-sharing negotiations. It can be argued that, from a commutative justice point of view, it makes a difference whether compensation is demanded in exchange for traditional knowledge or for providing access to a plant growing on state territory. Whereas in the latter case extensive claims on genetic resources might be difficult to legitimize, this is different for the case of traditional knowledge and likewise for domesticated plants. In these cases, the respective communities provide their intellectual good or the products of their work. This distinguishes their claims on this knowledge or plants from that of other communities. It can thus be reasoned that if communities provide such knowledge or breeding efforts, they have good reasons supported by commutative justice to demand compensation (Deplazes-Zemp, 2018a).

## Justice as Recognition

Justice as recognition is concerned with giving respect and recognition to every person.^10^ This means that each human being should be recognized for his or her equal moral status which at the same time implies that each person is respected for his or her individuality and differences to the norm (Taylor, 1992). Justice as recognition emphasizes that we *owe* recognition to others and that withholding recognition is a form of injustice. Feminists, racial movements, and multiculturalism are examples of political struggles for recognition. The importance of recognition has been supported by different philosophical arguments. For instance, Axel Honneth and Charles Taylor emphasize that recognition is a basic human need required for identity formation (Taylor, 1992; Honneth, 1995; Fraser and Honneth, 2003). Nancy Fraser suggests that lack of recognition violates the moral principle of “participatory parity” and is, therefore, unjust (Fraser, 2000; Fraser, 2001; Fraser and Honneth, 2003).

Justice as recognition is, besides distributive justice, another focus in the discourse on environmental justice (Schlossberg, 2007; Walker, 2012). As mentioned above, poor and minority communities often endure more environmental burdens such as waste dumpsites, toxic emissions, or other contaminations than privileged groups. In these cases unjust distribution usually occurs together with misrecognition of these groups who are being discriminated against and not given equal status in society. To achieve environmental justice, it is thus not enough to ensure fair distribution of risks and benefits; rather, it is also necessary to grant just recognition to members of different communities.

### Justice as Recognition for Genetic Resources

Even though in the philosophical literature on genetic resources the term “recognition justice” rarely appears, this dimension of justice also plays an important role.^11^ The framing of ABS as a system that mediates between providers of genetic resources in the “Global South” and users in the “Global North” indicates that the addressed injustice has also to do with political, economic and cultural power relations at the global level. Although the division between users in the “North” and providers in the “South” is an oversimplification, it is often used in the literature to refer to an unjust tendency. Industrialized countries often located in the “North” tend to profit from genetic resources, whereas biodiversity-rich countries mostly in the “South” are expected to protect genetic resources as biodiversity. This situation is not only unjust from the point of view of distributive justice and commutative justice as discussed above but the North–South interactions in the context of genetic resources also concern justice as recognition. This justice dimension is violated when the “North” takes advantage of historical power relations associated with economic and political privileges. In such cases, users of genetic resources tend to decide when and how to access which genetic resources without respecting providers as equal partners nor considering their customs, culture, or values. This type of misrecognition not only occurs in North–South interactions but also within provider states, when governments do not respected cultures or rights of minority groups. To prevent such misrecognition at global and local level, CBD, NP, and ITPGRFA explicitly acknowledge rights and claims of indigenous and local communities. It has been argued that more attention should be paid to justice as recognition in the context of biodiversity conservation to consider cultural differences (Martin et al., 2016; Robinson and Forsyth, 2016). For instance, certain communities object to the idea of patenting life (Tauli-Corpuz, 2003; Robinson, 2010). To offer them a share in benefits associated with patents on genetic resources misrecognizes their values and world view. Bram De Jonge discusses these issues under the heading of “cognitive justice,” which he defines as concerning the “recognition of the plurality of knowledge systems” (De Jonge, 2011: p. 135). I decided to use the concept of “justice as recognition” rather than “cognitive justice” not only because of the former’s philosophical tradition and its use in the environmental justice literature but also because, in my view, to recognize the individuality of others goes beyond acknowledging the cognitive aspect of different world views. The notion of recognition justice emphasizes the importance of a respectful attitude by the more powerful. As mentioned above, a similar idea is found in the notion of upstream benefit-sharing, which Bram de Jonge and Michiel Korthals include in their broader conception of distributive justice (De Jonge and Korthals, 2006; Korthals and De Jonge, 2009).

## Reparative Justice

I use the term “reparative justice” for the notion that redress is owed to those who suffered injustice in the past. In the literature, this dimension of justice is discussed under different related terms with sometimes overlapping and sometimes clearly distinct meanings (Daly and Proietti-Scifoni, 2011). Particularly in the legal context, the concept “retributive justice” is used for just punishment of those who committed injustice (Boersama, 2011; Daly and Proietti-Scifoni, 2011). This concept thus concentrates on the appropriate punishment of the offenders for the wrongs that they committed. In contrast, the term “corrective justice” usually refers to a conception of justice that focuses on the victims and on the just compensation that they should receive for suffered harms (Urban Walker, 2006). There are certain similarities between corrective justice and commutative justice. Both are linked to reciprocity, and both deal with just compensation. However, while the former concerns simultaneous exchange procedures, the latter is backward-looking and concerns compensation for something that happened in the past. “Restorative justice” is a third reparative justice concept besides “retributive justice” and “corrective justice.” Authors who use this concept highlights that righting injustice requires more than the remedy of harms: the important aim is to re-establish the relationship between victim and offender, usually in a relatively informal process, and to prevent that same type of injustice recurring in the future. Criminal trials are typically not seen as apt processes to establish restorative justice. Instead, reconciliation or mediation procedures are implemented in which offenders and victims meet, find the truth, and negotiate potential reparations (Johnstone and Van Ness, 2007; Marshall, 1999). Reparations in this sense are not necessarily material but can also consist of a formal apology (Urban Walker, 2006; Sharpe, 2007). A famous example of such a procedure was the Truth and Reconciliation Commission that was set up to address injustices committed during the apartheid era in South Africa. The importance that is given to establishing a relationship of mutual respect connects to “justice as recognition” described above. This illustrates once more how the different dimensions of justice overlap.

When I refer to “reparative justice,” I use it as an umbrella term to cover retributive justice, corrective justice, and restorative justice,^12^ with the idea that all of these aspects may play a role in righting injustice that took place in the past. In the environmental context, reparative justice, for instance, plays a role in climate justice, when it comes to the “polluter pays” principle (Caney, 2006; Caney, 2010). In Simon Caney’s words, this principle suggests that “those who caused a problem[ … ] should foot the bill” (Caney, 2006: p. 752). This can be understood as a demand for corrective justice, meaning that the polluters are responsible for repairing the damage that they caused. Maybe the “polluter pays” principle could also be interpreted as an example of retributive justice, particularly when one of the discussed objections to the principle is that the polluters were not aware of the effects of their actions and thus should not be the ones who pay (Caney, 2010). Based on that view, it would not be just to *punish* people for something that they caused unintentionally.

### Reparative Justice for Genetic Resources

Reparative justice is *not *a predominant justice dimension behind the notion of ABS for genetic resources. Doris Schroeder and Balakrishna Pisupati suggest that while distributive and commutative justice are directly relevant for ABS, reparative justice only plays a role in cases of non-compliance with the protocol which would trigger punishment and repair measures (Schroeder and Pisupati, 2010). However, the reparative justice dimension may still be relevant to understand why and how ABS systems have been implemented. The aim and need to redress some of the historical wrongs that were perpetrated—generally speaking—by the “Global North” against the “South” could be an underlying political motivation for establishing ABS systems. During colonial times, citizens of many biodiversity-rich states in the “Global South” suffered exploitation, oppression, subordination and disrespect, which amounts to injustice at the distributive, commutative and recognition dimensions. It cannot be denied that today colonial-like power relations still persist when the “North” exerts its strong political, cultural, and economical influence on the “South” accompanied by the same type of justice violation known from colonial times. From such a perspective, acknowledging biodiversity-rich states’ sovereignty over genetic resources could be understood as an acknowledgment of past misconduct as well as a commitment to consider the interests of less affluent countries in the future. That ABS systems are related to historical injustice has also been described by Jorge Cabrera Medaglia when he wrote: “The roots of ABS can be traced to colonialism and efforts by colonial powers to gain control of the trade in key commodities such as rubber, tea, and cinchona for their own benefit, with little regard for the communities and economies from which these resources originated” (Medaglia, 2015: p. 196). Moreover, Elisa Morgera highlighted that reparative justice played a role in the negotiations of the NP, when the African group demanded that benefit sharing should be extended to genetic resources which are available in *ex situ* collections, i.e., ones that had been exported from the country of origin in the past (Morgera, 2015: pp. 11–12).

## Procedural Justice

The term “procedural justice” has been used in a wider and a narrower sense. According to the former, this dimension of justice is concerned with any type of procedure that leads to a just outcome judged by criteria of other (substantive) justice dimensions. The narrower sense of procedural justice plays an important role in environmental justice, where procedural justice is discussed as the third central justice dimension besides distributive justice and justice as recognition (Schlossberg, 2007; Walker, 2012; Bell and Carrick, 2018). In this narrower sense, procedural justice concerns decision procedures and is achieved if the voice and interests of all involved parties are being considered. Because environmental justice was first the program of a social movement before it became a theoretical field of study, this procedural aspect has been particularly pivotal. One of the main aims of the movement was the empowerment of affected communities. Participatory approaches as a means for just procedures in decision making thus play an important role.

As for other justice dimensions different principles of justice have also been suggested for procedural justice. Derek Bell and Jayne Carrick distinguish three conceptions of procedural justice in the environmental justice discourse, which focus on three alternative principles (Bell and Carrick, 2018). The first principle is *political equality* according to which all affected parties should have an equal voice in the sense of equal power in environmental decision making (Bell and Carrick refer to: Shrader-Frechette, 2002). As an alternative, the principle of *proportionality* emphasizes that power in decision making should reflect the relative stake of the involved parties in the outcome of the decision (Bell and Carrick refer to: Bell and Rowe, 2012). The third conception of procedural environmental justice introduced by Bell and Carrick is based on the principle of *plurality*. Advocates of this principle criticize procedural justice conceptions emphasizing equality because they suppress difference, but it is argued that just procedures should consider differences in perspectives or interests. This is the same argument that is invoked in the literature on justice as recognition. Although the three principles for just procedures are discussed as three alternative ideals in the theoretical discourse, in practical environmental decision making they are combined in participatory approaches (Bell and Carrick, 2018).

### Procedural Justice for Genetic Resources

A focus on fair decision procedures is particularly relevant in the context of genetic resources because, as mentioned earlier, political and economic power between providers and users are often unequally distributed, which is a violation of recognition justice and/or distributive justice. Just decision procedures are thus not only an aim in themselves but also an approach to achieve more justice along other dimensions. The NP addresses some of these problems, for instance by highlighting rights of indigenous and local communities or by requesting scientific and technological collaboration (NP, Article 23). Moreover, one article of the NP is dedicated to capacity-building, it requires that all parties should cooperate in the endeavor of ensuring that also less affluent parties have the capacities to engage in the outlined ABS procedure (NP, article 22). Prior Informed Consent (PIC) and Mutually Agreed Terms (MAT) are two procedural requirements in the NP. Users of genetic resources must obtain PIC from providers.^13^ In response, the providers can authorize access to the resource under the condition that MAT are negotiated. Besides the conditions for the access to genetic resources MAT also determine the benefits that will be shared (Greiber et al., 2012; Biber-Klemm and Martinez, 2016). From a procedural justice point of view, these two elements should be implemented with a focus on the principles of political equality, proportionality and/or plurality. To what extent the procedure is participatory and considers these principles, particularly also concerning involvement of indigenous and local communities, depends not only on the users but also the provider states. Therefore, there is a considerable leeway to consider more or less procedural justice within the legal framework.

## Justice Challenges in Using Genetic Resources

In the article so far, I have attempted to show that all of the introduced dimensions of justice play a role in the context of ABS for genetic resources. In the following, I will discuss three particular justice challenges that arise in the use of plant genetic resources: first, biopiracy; second, environmental burdens from biodiversity conservation; and third, plant genetic resources in agriculture. These examples should illustrate the role that different justice dimensions play in the discourse and how they are being addressed or overlooked. In the course of this analysis, it will become evident that justice challenges must be addressed at two different levels: on the one hand the* institutional level*, which is represented by national and international policy; and on the other hand, the *individual project level*, which concerns specific projects of resource use with their respective ABS negotiations.

## Challenge 1: Biopiracy

To explain which types of injustice the ABS scheme of the CBD addresses, authors frequently invoke the problem of biopiracy (e.g. Kamau et al., 2010; Millum, 2010). The term refers to unauthorized use of genetic resources or traditional knowledge in the development of a product. Those accused of biopiracy did not share any benefits, recognition or material profit with the community that provided the resources or knowledge in question. Particularly when traditional knowledge is involved, biopiracy is a violation of commutative justice because the providers of this knowledge made an essential contribution to the final product, for which they were not compensated. However, biopiracy is not only a violation of commutative justice. When traditional knowledge was used without the consent of the community, justice as recognition was also violated, because the members of the community were not respected as equal negotiation partners and their rights to their cultural heritage were not acknowledged. One of the aims of ABS in the CBD and NP was to address this type of commutative injustice and misrecognition by introducing just procedures with the requirement for PIC and MAT not only for the export of genetic resources but also for the use of traditional knowledge.

However, using the famous case of the Hoodia cactus, Saskia Vermeylen and Gordon Walker showed that the implementation of an ABS agreement alone does not automatically warrant recognition justice or procedural justice. The succulent plant *Hoodia* *gordoniiwa* was used as an appetite suppressant by the San, a hunter-gatherer community in southern Africa. In 1996, scientists of the South African Council for Scientific and Industrial Research (CSIR) isolated and patented the active compound of *Hoodia*, without involving the San or acknowledging their active contribution. Eventually, under external pressure, the CSIR eventually got in touch with the San, recognized their contribution, and in 2003 the CSIR and the South African San Council signed a benefit-sharing agreement (Beattie, 2005; Wynberg, 2005; Vermeylen and Walker, 2011). Even though the CSIR obtained PIC by the official representatives of the San, Vermeylen and Walker doubt that this was a case of procedural justice. In some places, the process was not really participatory because most group members were not aware of it. Moreover, the San did not receive enough legal and strategic assistance in the negotiation process and their opinion was not really taken into account. Vermeylen and Walker further cite the Hoodia Benefit-Sharing agreement as a case of violation of justice as recognition, which requires not only recognizing the other party as a partner with equal status and rights but also acknowledging and considering differences in culture and political tradition (Vermeylen and Walker, 2011). The San are known as an egalitarian community that functions without formal political institutions and power hierarchies, but with decision making processes that involve consensus procedures. Even if the PIC and MAT procedures had been performed according to liberal democratic Western ideals, they would have been inflicted on this community against its own political tradition and values, because only a selection of representatives where involved in the negotiations.

Finally, in the *Hoodia* example, reparative justice also plays a role. As mentioned above, the CSIR originally patented the active compound in *Hoodia* without involving the San, and it acknowledged their contribution only under external pressure. The San thus had been wronged and the question is whether it is enough to ensure commutative, recognition and procedural justice in the future or whether, in addition, material or symbolic reparation would be appropriate.

## Challenge 2: Environmental Burdens From Biodiversity Conservation

The second justice challenge in the context of plant genetic resources concerns the background against which the ABS system has been developed in the CBD. The CBD has three main objectives, first, the conservation of biodiversity; second, the sustainable use of its components; and third, the fair and equitable sharing of benefits from genetic resources. The first objective places high demands on biodiversity-rich countries, which at the same time are often low-income countries in the “South” that struggle with poverty. The second objective, too, addresses particularly this group of countries, because industrialized countries in the “North” already went through industrial development, albeit in a non-sustainable way. It thus seems that the “Global South” carries a disproportionate burden when it comes to biodiversity conservation, even if affluent countries cover some of the conservation costs. Biodiversity-rich countries are affected, for instance by opportunity costs resulting from the requirement to setup protection areas which implies that communities living on that land waive the right to cultivate it. Other burdens associated with conservation projects include the displacement of local communities to restrict human influence on the respective area (Agrawal and Redford, 2009). In that sense, the burden of biodiversity conservation is not distributed justly. What makes this distributive injustice more pronounced is that benefits from the use of genetic resources, directly attributable to the effort of conserving biodiversity, tend to flow into the “Global North”. The request to ensure that the “South” also gets a share in benefits could thus be understood as a means to achieve more distributive justice. I suggest elsewhere that mitigation of the unfair distribution of burdens and benefits from biodiversity could be linked through a global biodiversity fund, which would be supported by those who benefit from genetic resources and would be disbursed for biodiversity conservation projects (Deplazes-Zemp, 2019). Interestingly, the Multilateral System of the ITPGRFA has established a similar type of fund financed by benefits from genetic resources and is used to support projects in conservation or development of agriculture. However, as will be elaborated below, the fund of the Multilateral System was not primarily set up with the aim to ensure fair distribution of the burden of biodiversity conservation but to ensure food security. These distributive justice issues could be implemented at the institutional level, but when it comes to individual ABS negotiations, genetic resource users could also acknowledge this issue, for instance by contributing directly to justly designed biodiversity conservation projects in the provider country.

The idea that those who profit from biodiversity, for instance in bioprospecting projects, contribute to biodiversity conservation can also be understood as a requirement of commutative justice. Based on this view, the contribution would be understood as a form of compensation for the possibility to benefit from genetic resources, which persisted thanks to biodiversity conservation (Deplazes-Zemp, 2018a).

Justice as recognition is important in this context too. It is necessary to recognize and consider the differing values, norms and needs of the contracting parties to be able to understand burdens associated with biological conservation, as well as the benefits that could be shared. Being open to other world views may involve, for instance, calling into question our own understanding of biodiversity and nature as opposed to humanity (De Jonge, 2011; Martin et al., 2013).

Finally, at least at the institutional level the dimension of reparative justice also plays a role when it comes to the connection between ABS for genetic resources and environmental burdens. That biodiversity is threatened today is to a large degree a consequence of the lifestyle and unsustainable development in the “Global North”. With reference to reparative justice, one might thus reason for a “destroyer pays” principle analogous to the “polluter pays” principle in the context of climate justice. Moreover, biodiversity conservation may also have a colonial legacy, because important national parks were established for the benefit of colonial rulers without any consideration for traditional culture and lifestyle (Chan and Satterfield, 2013). Even if current ABS negotiations may not be the right occasion to repair these historical injustices, awareness and acknowledgment of the need for reparative justice may help to understand and respect the situation of communities and states in biodiversity-rich regions.

## Challenge 3: Plant Genetic Resources in Agriculture

Plant genetic resources used in agriculture pose additional justice challenges to other types of genetic resources. In the following I will emphasize the role of the different dimensions of justice in these particular challenges. One of the differences between plant genetic resources in agriculture and, for instance, genetic resources of plants in the rainforest, is that agricultural plants are not purely natural (e.g., Halewood et al., 2013; Deplazes-Zemp, 2018b). They are domesticated, meaning that they are the result of breeding processes in which humans shaped crops over centuries. In contrast to purely natural resources, domesticated plants were generated by certain communities, farmers, and breeders or more recently by scientists in companies, who thus have particular claims on these plants. In that sense, a demand to distribute benefits from these resources equally among everybody (those who contributed to the plant and those who did not do so) seems to be more difficult to legitimize. Farmers or breeders who “improved” these plants can appeal to commutative justice and argue that if they make these plants available, their effort and creativity should be acknowledged and compensated (Deplazes-Zemp, 2018a; Deplazes-Zemp, 2018b). Intellectual property rights (IPR), such as patents and plant breeders’ rights, have been used as mechanisms to implement such compensation by ensuring that only the holders of these rights are entitled to commercially benefit from the plant in question. Although these property rights may be well suited to acknowledging the contribution of scientific innovation of commercial breeders in industrialized countries, they are unsuitable to account for the collective contributions of small-scale farmers in low-income countries, where the use and generation of novel varieties go hand in hand and cannot be assigned to individual breeders who put the crop on the market at one particular moment (Borowiak, 2004; Correa, 2015; Oguamanam, 2018; Adebola, 2019). IPR over plant genetic resources, thus, raise a variety of justice issues. They fail to achieve commutative justice because farmers with particular claims on these resources cannot profit from the IPR system. Moreover, this is also a case of misrecognition. IPR fail to take into account agricultural practices of farmers in low-income countries. Consequently, not only the particular contributions of these farmers to valuable agricultural plants are being misrecognized but also the claims that these communities have on their products and their particular interests to be compensated if their products are being used. To correct these commutative and recognition injustices toward small-scale farmers, the concept of “farmers’ rights” was brought into the discourse to account for the generation of new varieties by farmers and grant them certain rights over these plant genetic resources (Borowiak, 2004). The ITPGRFA was the first international binding treaty that explicitly recognized farmers’ rights; in addition, the literature also discussed ABS as outlined in the CBD and NP as an approach that allows accounting for farmers’ contributions *via* PIC and MAT (Correa, 2015; Oguamanam, 2018; Adebola, 2019).

How different dimensions of justice come together and may lead to different expectations in case of plant genetic resources in agriculture can also be illustrated with the previously mentioned Multilateral System of the ITPGRFA. The Multilateral System provides facilitated access to 64 of the most important crops and requires that those who accessed genetic resources through this system will freely share resulting benefits or pay a percentage of their profits into a common fund.^14^ Agricultural plant genetic resources covered under the Multilateral System can be accessed without obtaining PIC and negotiating MAT but by using a standard template the “Standard Material Transfer Agreement” (SMTA) negotiated by the Parties to the ITPGRFA. However, since benefits from the use of these resources do not flow to the provider but to the Multilateral System as a third party, none of the contracting parties have a particular interest in monitoring compliance with the SMTA. To address this problem, the concept of the “Third-Party Beneficiary” has been introduced into the SMTA. The parties to the SMTA agree that the Third-Party Beneficiary has certain rights, for instance, to monitor compliance with the agreement (Manzella, 2013; Moore, 2013).

Analyzed from the stance of the different dimensions of justice, the problem of compliance with the SMTA arises because of the combination of two justice dimensions. An agreement between providers and users of genetic resources usually aims at implementing commutative justice by ensuring that the exchange is fair. In contrast, the Multilateral System supports distributive justice ensuring that the possibility to access and benefit from these resources is distributed fairly and recognition justice in the sense of recognizing basic rights to food. These aims can, however, not be secured by a procedure that involves only two “self-interested” negotiation parties. The introduction of the Third-Party Beneficiary can thus be understood as a means ensure distributive or recognition justice in a commutative justice framework.

As just indicated, the Multilateral System points to another, more fundamental, ethical challenge raised by agricultural plant genetic resources, which has to do with the role of agriculture in the production of staple foods and in ensuring food security (De Jonge and Korthals, 2006). Safeguarding this role of agricultural genetic resources was the main motivation to provide facilitated access to the 64 crops covered under the Multilateral System. Food is a basic good that people need for health and well-being, therefore, an understanding of humans as beings with basic moral rights implies the duty of ensuring food security. This aim goes beyond what is demanded by a classical natural resource oriented understanding of distributive justice.^15^ While the latter demands fair distribution of *existing* goods, the basic moral right to food necessitates safeguarding that enough food is available. This can lead to a positive duty to ensure that food can be produced also, for instance, under changed climatic conditions.^16^

Recognition justice is also relevant in this context, when it is demanded that every person’s moral right to food must be acknowledged with consideration for varying needs and interests. Bram de Jonge pointedly describes how there is a certain type of tension between this basic moral requirement of ensuring food security^17^ and commutative justice (De Jonge, 2011). Although the first moral requirement suggests that farmers or breeders who develop new staple foods should make them available to everybody, the latter demands that they be compensated for their products. However, from the identification of such a tension it does not follow that one of the moral requirements must be prioritized. Instead, the situation can be understood as the challenge to find solutions to implement both moral demands. This challenge cannot only be addressed at the institutional level by drafting all-encompassing regulations; it must also be addressed at the individual project level, by acknowledging and rewarding contributions to agricultural plants even when this may not be explicitly required by the regulatory scheme.

The purpose of this section was to illustrate that usually more than one justice dimension is concerned in justice challenges involving plant genetic resources. This insight can be practically relevant for at least three reasons.

First, too narrow a focus on one dimension of justice may lead to unsatisfying results, because injustice at other dimensions persists, for instance, if biopiracy is seen exclusively as a violation of commutative justice and aspects of justice as recognition or reparative justice are being ignored. Although PIC and MAT may suffice to warrant commutative justice as defined in a Western context, justice as recognition requires considering potential conflicts of values between the involved parties. Consequently even our own principles of procedural justice may need reflection as described in the case of negotiations with the San above. The problems with benefit sharing as part of the Multilateral System also indicated that the focus on only one dimension of justice is not sufficient. A procedure that was constructed to deal with commutative justice needs adaptation to be able to address aims of distributive justice or recognition justice.

Second, tensions may arise between the different justice dimensions. For instance, certain parties highlight that plant genetic resources in agriculture are cultural products and those who developed them have special claims on benefits, which need to be considered by commutative justice. Others, however, highlight that as resources relevant for food security, plant genetic resources should be freely available and subject to distributive or recognition justice. The previously mentioned biopiracy case involving the San also reveals tensions between an ideal of procedural justice and justice as recognition. In certain cases, this type of tension may lead to difficult ethical dilemmas, for instance, when a community for traditional reasons does not agree to involve women or young people in decision procedures. Should their culture be respected or should the participatory ideals of procedural justice be implemented?^18^

Third, the focus on different justice dimensions may lead to different expectations in negotiation processes. In the discussed biopiracy case, the companies understood their task in the first place in the sense of commutative justice as providing compensation. However, the San also expected to be recognized. Although officially ABS systems are drafted to achieve commutative justice at the level of individual projects, certain stakeholders also seem to expect that such a system establishes distributive justice at a global and international level.

## Conclusions

In this article, I introduced five dimensions of justice that play a role in dealing with plant genetic resources in “wild” nature and agriculture, namely distributive justice, commutative justice, justice as recognition, reparative justice, and procedural justice. I then analyzed how these dimensions meet and overlap in some of the major justice challenges faced in the context of plant genetic resources. The article aims at giving some insight into why the use of genetic resources generates so much controversy and into what needs to be considered in regulating and handling them justly. I will respond to these aims with two conclusions for a just use of plant genetic resources.

First conclusion: Justice challenges posed by the use of plant genetic resources are multi-faceted and cannot be addressed by focusing exclusively on one dimension of justice. The analysis of the literature shows that different authors concentrate on different justice-related questions raised by genetic resources. I presented these different questions along the five introduced dimensions of justice. They often overlap, but to reduce the justice challenges to one or two of these dimensions would not do justice to the complexity of practical issues. Assuming that all of the justice dimensions are strong moral demands, we thus need to account for each dimension and consider how we can address them together when we work toward more fairness and equity in dealing with plant genetic resources.

Second conclusion: Because justice challenges are multi-faceted, they cannot be met at the institutional level alone, but the challenges must be identified and addressed for each case individually. This overview article indicates that existing institutional ABS frameworks can be used to address several of the justice challenges, but at the same time these regulatory frameworks seem not sufficient to meet the challenges. Therefore, it is particularly important that the individual users of genetic resources, including researchers who work in non-commercial projects, are also aware of the issues at stake and find individualized solutions to address them. There is a risk that the increasing administrative burden associated with ABS obscures the actual justice issues at stake. However, losing sight of these issues at the project level would certainly work against the aim of increasing fairness and equity in ABS for plant genetic resources.

## Author Contributions

AD-Z wrote this article alone and is responsible for the literature search, the structure and design of the article, as well as the content and argumentation.

## Funding

This work was supported by the UZH Research Priority Program (URPP) on “Global Change and Biodiversity.”

## Conflict of Interest

The author declares that the research was conducted in the absence of any commercial or financial relationships that could be construed as a potential conflict of interest.

## Footnotes

1 Even though justice in this sense requires considering other worldviews as part of doing justice to other communities, it cannot be denied that the approach itself is driven by a particular Western tradition of thought. It would be beyond the scope of this article to consider different cultural approaches to justice, but this work may serve as a starting point for a wider intercultural comparison. Moreover, I would like to clarify that this is not a work in legal theory, which interprets justice within a legal framework but rather in moral philosophy, reflecting on how the legal framework should be constructed in order to be able to respond and solve ethical challenges of justice.

2 The text of the Convention on Biological Diversity is available at: https://www.cbd.int/convention/text/ (accessed May 2019).

3 The text of The Nagoya Protocol is available at: https://www.cbd.int/abs/ (accessed May 2019).

4 The text of the International Treaty on Plant Genetic Resources for Food and Agriculture is available at: http://www.fao.org/plant-treaty/overview/texts-treaty/en/ (accessed May 2019).

5 The five dimensions of justice should be understood as heuristic groups, which serve the aim of facilitating the analysis of justice questions that arise. I am not aiming at developing a theory of justice, I do thus not rank the dimensions of justice; none of them is treated as having general priority over the other. Moreover, I do not take any position as to whether one justice dimension is generally primary to another one. Therefore, I do, for instance, not argue in favour of or against Axel Honneth’s position according to which unjust distribution is a result of misrecognition (Fraser and Honneth, 2003). I acknowledge, however, that the different dimensions of justice can overlap. An unjust situation can concern more than one justice dimension. In the context of environmental justice, Gordon Walker, for example, highlighted that different dimensions of justice are often closely linked, procedural injustice can be an explanation for distributive injustice (Walker, 2012: p.47); then again, unjust distribution of power and money or misrecognition of certain communities can be a cause for unjust procedures.

6 Although many authors acknowledge the informational nature of genetic resources (e.g. Vogel, 1994; Millum, 2010; De Jonge, 2011; Tvedt and Schei, 2013; Ruiz Muller, 2015; Deplazes-Zemp, 2018b), the definition of genetic resources in the CBD refers to „genetic material of actual or potential value“ and there is some controversy on how this should be interpreted (Tvedt and Schei, 2013).

7 Annex to the Nagoya Protocol (p. 24) available at: https://www.cbd.int/abs/ (accessed May 2019).

8 The “fair and equitable” clause could be read as another indication of a connection to commutative, since the term “equity” has been used to describe the aim of commutative justice (Adams, 1963; Cook and Hegtvedt, 1983).

9 The idea that those who use information in nature should pay compensation for the resulting benefits indicates that there is another justice aspect that could be considered in the context of plant genetic resources, namely ecological justice. Nicholas Low and Brendan Gleeson contrast ecological justice with environmental justice by explaining that the latter deals with “conflicts among humans over nature” whereas ecological justice deals with conflicts “between humans and nature” (Low and Gleeson, 1998: p. 49 emphasis in the original). Ecological justice is thus concerned with justice towards the non-human world (Schlossberg, 2007) which is beyond the scope of this article. Whereas on an ecological justice perspective, just compensation for information in nature would require giving something back to nature, I understand just compensation for natural information here as a compensation for those human communities who actively protect biodiversity.

10 It might be objected that when I defined justice in general as meaning to “acknowledge that all human beings have equal moral status and basic moral rights” (see above) this already involves recognition, which thus is a precondition of all dimensions of justice. It is true that in order to be able to enter a justice relationship with someone and to identify injustices of any type I must recognise the other as having moral status and basic rights. However, there are cases of injustice, where none of the other justice dimensions have been violated but the injustice lies in a form of misrecognition, for instance cases where someone’s belief, culture or the like are not being respected. These cases where misrecognition per se is the injustice are discussed here under the heading of “justice as recognition.” The possibility that some form of basic misrecognition also underlies other types of injustice does not make it redundant to introduce a separate justice dimension of justice as recognition in order to account for the different types of injustice that can occur in practice.

11 The following articles are exceptions in the sense that ABS is explicitly discussed in relation to environmental justice including recognition justice: (Vermeylen and Walker, 2011; Martin et al., 2013; Morgera, 2015; Martin et al., 2016).

12 Some conceptions of restorative justice seem not to be covered by my concept of “reparative justice”. For instance, what Johnstone and Van Ness call, the “encounter conception of restorative justice”, which emphasizes the importance of the encountering process rather than the aim of redressing those who suffered injustice (Johnstone and Van Ness, 2007).

13 PIC implies that before accessing the resource in question, the potential user must provide all the relevant information on the intended utilization project, including the conditions of access as well as the intended use.

14 The Multilateral System: http://www.fao.org/plant-treaty/areas-of-work/the-multilateral-system/overview/en/ (accessed May 2019).

15 This may not apply to a less traditional conception of distributive justice associated with the notion of “upstream benefit-sharing” suggested by Bram de Jonge and Michiel Korthals. According to this understanding distributive justice needs to focus on a globally more just distribution of opportunities to set the research and innovation agenda. This could lead to a prioritization of the positive duty of ensuring that food resources are being generated where they are scarce (De Jonge and Korthals, 2006; Korthals and De Jonge, 2009).

16 An according duty could have been a motivation to facilitate, and thereby encourage, access to the 64 most important crops for farmers, breeders and researchers in the Multilateral System.

17 De Jonge speaks of a “principle of need and equity” (De Jonge, 2011: p137f).

18 Usually, it is held that recognition justice cannot justify the violation of basic human rights. In that sense, the United National Declaration on the Rights of Indigenous Peoples (UNDRIP) also requires that indigenous peoples’ institutional structures and their procedures need to be “in accordance with international human rights standards” (UNDRIP, article 34, available at https://www.un.org/esa/socdev/unpfii/documents/DRIPS_en.pdf (accessed August 2019)).
